# Multi‐method approach to assessing the floral‐visiting insect assemblage of rare, ambophilous plant *Baccharis vanessae* in Southern California

**DOI:** 10.1002/ece3.70327

**Published:** 2024-10-22

**Authors:** Christina Simokat, Elizabeth L. Ferguson, Jessica Keatly, Tyler Smith, Mia Lorence, Jasmine O'Hara

**Affiliations:** ^1^ California State University, San Marcos San Marcos California USA; ^2^ San Diego Pollinator Monitoring Program Encinitas California USA; ^3^ Ocean Science Analytics LLC San Diego California USA; ^4^ San Diego Botanic Garden Encinitas California USA

**Keywords:** camera traps, conservation management, focal observations, insect–plant interactions, plant conservation, pollination, video observations

## Abstract

Insects are the major pollination vectors for angiosperms, and insects native to a given habitat can play an irreplaceable ecological role in food webs and plant reproduction. With precipitous declines in insect species over the last decades, it is urgent to document insect assemblages in native plant communities to support conservation efforts. Identifying pollinators and their pollination activity is challenging; however, emerging technological methods are providing new monitoring capabilities. In this study, we compare the accuracy of two different methods of monitoring to assess the flower‐visiting insect assemblage and likely pollinators of Encinitas baccharis (*Baccharis vanessae*): focal observations and video recordings from camera traps. *B. vanessae* is a rare, endemic species found in Coastal Sage Scrub communities in San Diego County. This federally listed species is threatened by habitat loss and fragmentation, which may also be affecting the availability of its insect pollinators. Results indicate that *B. vanessae* supports and is supported by a variety of flower‐visiting insect groups. The diversity of insect visitors at male and female plants were similar across all diversity measurements. The insect vectors identified were as expected given *B. vanessae* pollination syndrome. This syndrome also aligns with wind as a pollination vector, providing evidence of ambophily. While focal observations underreported insect activity by approximately half, the proportions of common diurnal visitors were similar with both methods. Camera traps were unable to provide sufficient detail to discern visually similar groups, but were able to record nocturnal insect activity, which was dominated by moths (Lepidoptera, 82%). While collection protocol in this study did not record the time an insect spent interacting with a flower, we anecdotally observed moths spent notably longer periods in contact with flowers than most diurnal insects. This study has implications for effective monitoring and conservation of endangered plant species and their affiliated pollinators.

## Graphical abstract

In this study, we compare the accuracy of two different methods of monitoring to assess the insect assemblage of Encinitas Baccharis (*Baccharis vanessae*): focal observations and video recordings from camera traps. The insect vectors identified were as expected given *B. vanessae* pollination syndrome. While focal observations underreported insect activity by approximately half, the proportions of common diurnal visitors were similar with both methods. Camera traps were unable to provide sufficient detail to discern visually similar guilds, but were able to record nocturnal insect activity, which was dominated by moths (Lepidoptera, 82%).

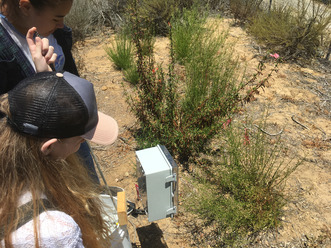

## 1 INTRODUCTION

Pollinators are in decline globally, yet the survival of native habitats are dependent on pollinators (Hoffman Black et al., 2008; Potts et al., 2010). Pollination is essential for plant reproduction, and 75% of flowering plants require animal pollination (Ollerton et al., 2011). Insects are the major pollination vectors for angiosperms (Winfree et al., 2011) and they are also integral to the food web of most habitats, feeding more than 80% of bird families as well as mammals, amphibians, and reptiles (Klasing, 1998). Insects that are native to a given habitat can play an irreplaceable ecological role in food webs and plant reproduction (Pellmyr et al., 1996; Suarez et al., 2000; Travis & Kohn, 2023). As many insect species have seen precipitous declines over the last decades, it is urgent to document insect assemblages in native plant communities to support conservation efforts.

San Diego County is an area of especially high diversity within the California Floristic Province, which is one of the global biodiversity hotspots; however, little is known about the pollinator networks in native habitats (Dartnell et al., 2022; Jennings et al., 2018; Stein et al., 2000). Coastal sage scrub (CSS) comprised of low‐growing, soft‐woody sub‐shrubs typically below 3000′ elevation, can often be found adjacent to or mixed with chaparral vegetation, particularly in xeric locations (Westman, 1981). CSS plants are often characterized as sclerophyllous, with small leaves and flowers (Westman, 1981). Historically, CSS comprised 2.5% of natural habitat in the state of California (Westman, 1981). Habitat destruction and fragmentation due to development have resulted in 85%–90% loss in <100 years (Riordan & Rundel, 2014; Westman, 1981). CSS has been designated critical habitat for the endangered California gnatcatcher (Fish and Wildlife Service, 2000) and contains many endemic species and more than 100 rare species of plants and animals (Dixon, 2003).

While anemophily, or wind‐mediated pollination, is the most common abiotic pollination mode, in CSS habitats as well as among all angiosperms, biotic pollination is far more common and effective (Moldenke, 1976; Willmer, 2011). Anemophilous pollination syndrome traditionally includes small, pale colored flowers with little or no scent or nectar in plants that are often dioecious and associated with a dry climate with moderate wind (Willmer, 2011). However, these characteristics are similar to pollination syndromes associated with entomophily, including functional insect groups such as beetles, flies, moths, and small bees, as well as the generalist small insect‐pollination syndrome described by Bawa et al. (1985). Strict anemophily is considered uncommon in chaparral and CSS plant species, despite the associated traits and climate being present (Moldenke, 1976). Rather, these habitats are known for abundant flower‐visiting insects, and San Diego County has some of the highest bee species richness in the world (Hung et al., 2015; Moldenke, 1976). Ambophily may be defined as the regular pollination of a plant by both wind and insects leading to greater reproductive success than if either mode was utilized alone (Abrahamczyk et al., 2023). It is not clear whether ambophily is a pollination mode or a pollination syndrome, or an evolutionary transition between anemophily and entomophily. However, for most ambophilous plants, insect pollination is more important than wind pollination for reproductive success and genetic diversity, and therefore, it is vital to identify insect pollinators for conservation (Abrahamczyk et al., 2023; U.S. Fish and Wildlife Service, 2011).

### 1.1 Determining the insect pollinator assemblage of *Baccharis vanessae*

Encinitas Baccharis (*Baccharis vanessae* R.M. Beauchamp) is one rare, endemic species found in CSS and chaparral communities along the coast and a few interior areas of San Diego County (U.S. Fish and Wildlife Service, 2020). The rangewide population is estimated to be <2000 individuals, all of which were occurring in maritime climate at 900 m or lower elevation. It is a dioecious shrub 0.5–1.3 m tall, with small white to pale yellow flower heads 2.5–4 mm long (Beauchamp, 1980; U.S. Fish and Wildlife Service, 2011). Federally listed since 1996, this species is threatened by habitat loss and fragmentation, which may also be affecting the availability of its insect pollinators (U.S. Fish and Wildlife Service, 2011). Previous research has shown it to be both wind and insect pollinated, but specific insect pollinators have not been identified (San Diego Management & Monitoring Program, 2010).

Conservation efforts have been limited and not highly successful (San Diego Management & Monitoring Program, 2010). Remaining *B. vanessae* populations have stayed small over the years it has been protected, and this low recruitment rate and low reproductive output may be connected to smaller numbers of insect pollinators (Native Plant Program, 2015; San Diego Management & Monitoring Program, 2010; U.S. Fish and Wildlife Service, 2011). As genetic diversity in the populations is low, gene flow is occurring, even between distant sites (Milano & Vandergast, 2018). Flow could be attributed to insect interaction, but seed‐dispersal agents have also not been identified for this species (Native Plant Program, 2015; U.S. Fish and Wildlife Service, 2011). Given that transplantation of seedlings to new sites and long‐term storage of seed have been reported to have limited success in the past, and the declines in insect populations globally, more information about insect assemblages associated with *B. vanessae* is needed to successfully manage this threatened species (San Diego Management & Monitoring Program, 2010; U.S. Fish and Wildlife Service, 2011).

### 1.2 Multiple methods of determining pollinating insect assemblage

Despite the importance of pollination to heathy native plant communities, identifying pollinators and their pollination activity is challenging. Historically, insect monitoring has involved sweep netting, malaise, pan, vane and pitfall traps, beating sheets, and focal observations, also called active visual surveys (Montgomery et al., 2021; Prendergast et al., 2020). More recent methods involve the use of acoustic surveys and camera traps (Droissart et al., 2021; Gilpin et al., 2017). These methods can incorporate the use of machine learning detection or classification of insects in either static imagery or video data. Standardization of methods are under development; however, the diversity of insects, their habitats, and ecological roles, make it likely that multiple methods and technologies will be employed and improved in the near future (Montgomery et al., 2021). Alternate, reliable methods of observation are required for better understanding the temporal variation and taxonomic variation of insect–plant interactions.

Focal monitoring is widely used and involves a human researcher observing and recording insect interactions (O'Connor et al., 2019). While focal monitoring results may be limited by the level of expertise of the researcher in identifying insect species, it is nevertheless proven successful with even a small amount of training and it is cost‐effective and low to no impact on the study area (Gibson et al., 2011; Montgomery et al., 2021; Stroud et al., 2015).

Camera traps use video or photo stills to identify insects and offer significant research benefits. Recent advances in technology have provided low cost, high‐definition camera options that require minimal technical skill. Camera traps can be deployed so that they have little to no impact on plants or the soil around them, and they reduce the need for human trampling of plants and animals in the study area. Newer cameras provide flexible settings to accommodate different targets. Many cameras can be motion activated; however, this is not a reliable function for insect targets, as the motion may be too fast and small to trigger the camera, and in certain circumstances, the motion of the plant may cause false triggers (Naqvi et al., 2022). Time‐lapse video is an emerging method for classification and documentation of insect activity (Alison et al., 2022; Bjerge et al., 2022; Collett & Fisher, 2017; Edwards et al., 2015).

In the realm of pollinator research, several studies have explored innovative methods for studying insect interactions with flowers using video technology. Bonelli et al. (2020) proposed an integrated approach that combines manual sampling and video observations to comprehensively study flower‐visiting arthropods in high‐mountain environments, recognizing the importance of employing both methods to capture a holistic understanding of such interactions. Gilpin et al. (2017) directly compared focal and camera monitoring efficacy with different flower morphologies, demonstrating the limitations of camera use. Curran et al. (2020) introduced three‐dimensional videography as a nonlethal technique to enhance visual insect sampling, highlighting its potential to improve accuracy and efficiency in insect monitoring and research. In the context of bumblebee monitoring, Steen and Orvedal Aase (2011) developed a portable digital video surveillance system, demonstrating the application of technology in ecological research for monitoring flower‐visiting bumblebees. Azarcoya‐Cabiedes et al. (2014) explored automated video analysis for the detection of bumblebees, showcasing the possibilities of streamlining and enhancing pollinator tracking through automated techniques. These studies collectively contribute to the advancement of our understanding of insect pollinators and their interactions with flowers, employing video technology as a valuable tool in the field of ecological research.

The greatest advantage of camera traps, however, may be the ability to document nocturnal pollinators and general insect activity. Nocturnal focal observations are difficult, as using a light source sufficient for human sight would provide an artificial attractant for nocturnal insects, biasing the results. Nocturnal pollination, predominantly attributed to nocturnal Lepidoptera species, has been associated with increased gene flow between small populations of plant species (Barthelmess et al., 2006; Macgregor et al., 2015). A number of Lepidoptera species have been identified as having long migratory routes, indicating the ability to transport pollen—genetic material—between isolated populations (Macgregor & Scott‐Brown, 2020; Suchan et al., 2019). Modern camera devices typically incorporate infrared technology to assist in documenting nocturnal pollinators (Brehm et al., 2021; Walton et al., 2020). The combination of high resolution, night and daytime monitoring, and greater temporal resolution enable opportunities for better understanding pollinator activities. The high temporal resolution of video monitoring offers a unique opportunity for evaluating ecosystems on a small scale.

This study has two goals: we will (1) assess the flower‐visiting insect assemblage of *B. vanessae* observed in its 2023 bloom period, and (2) compare the accuracy of two different methods of insect monitoring: focal observations and video recordings from camera traps. By identifying the insect groups associated with *B. vanessae*, we can better target conservation strategies for its successful reproduction. This insect assemblage will contain pollinators, of varying efficiency. Some insects may only pollinate accidentally or ineffectively, but the relative contributions of each insect group observed was not determined in this study. We compare the observations collected during several monitoring periods to collocated video recordings. As a secondary, yet important, effort, we assess the utility of standard camera traps for nighttime recording of potential pollinators. The aim of this comparison was to evaluate the efficacy of this method for increasing the understanding of pollinator relationships with endangered plant species. These emerging methods have the potential to be applied to the conservation and management of rare plants.

## 2 MATERIALS AND METHODS

Sampling took place at the San Diego Botanic Garden (SDBG) in Encinitas, California, in the ‘Native Plants, Native People’ section. SDBG is a nonprofit public botanic garden that is located within historic CSS and Southern Maritime Chaparral habitat ranges and currently contains 11 acres of these plant communities (SDBG, n.d.). Reports from agencies vary but there are likely between six and 15 populations of *B. vanessae* remaining, and all but one are located in San Diego County (San Diego Management & Monitoring Program, 2010). SDBG has successfully established six plants at this site from seedlings—three male and three female. This population was established from seed collected from an extant wild population found <2 km to the southeast, and it is located approximately 1 km northwest from the site where the species was first described (Beauchamp, 1980; Gurnoe, 2021). Through a collaborative research partnership with SDBG, we were able to both observe the plant for focal observations and setup a wildlife camera that could be regularly accessed. We chose this site over the nearby wild population to avoid our cameras being stolen.

Monitoring began at the start of the bloom period, on July 16, 2023, continuing for 8 weeks until end of flowering was reached for all six plants by August 29, 2023. While the bloom period has been reported between August and November, our experience and consultation with SDBG horticulture staff indicated that *B. vanessae* can bloom at different times, and for shorter periods (Beauchamp, 1980; San Diego Management & Monitoring Program, 2010).

We used two primary sampling methods: focal observations and video data from camera traps. Focal monitoring consisted of 15‐min observations of one male and one female plant on the site with flowers in bloom between the hours of 9 a.m. and 6 p.m. Monitoring does not take place during adverse weather conditions for aerial insects, such as rain or strong wind. A single researcher stood adjacent to the plant, recording each pollinator interaction, which we define as an individual insect touching a flower in any way that it could gather pollen or deliver pollen. Contact of an insect with a flower's reproductive organs is a method of assessing pollinator effectiveness used in related studies of pollinator ecology (Rosas‐Guerrero et al., 2014). Insects were classified according to broad groupings (Table 1). We proposed to collect data for at least 50% of the blooming period, opportunistically when research associates were available at the *B. vanessae* site. All field data were digitally collected using Kobo Toolbox which allows for collection of monitoring data and images (Harvard Humanitarian Initiative, 2021). Focal observations were staggered during the day to allow for representation of morning, afternoon, and dusk insect visitor monitoring activity.

**TABLE 1:** Insect groupings used by the San Diego Pollinator Monitoring Program for collecting identification information.

Grouping code	Insect grouping	Functional grouping	Examples
ARAN	Araneae	Spiders	(Not insects)
ARMA	Isopoda: Armadillidiidae	Pillbugs	(Not insects)
COLE	Coleoptera	Beetles	Flower longhorn beetle (*Stenelytrana emarginata*), Tumbling Flower Beetle (Mordellistena spp.), Stink Beetle (Eleodes spp.), Darkling Beetle (*Apsena pubescens*), Black Rain Beetle (*Pleocoma puncticollis*), Asian lady beetle (*Harmonia axyridis*), and Convergent Lady Beetle (*Hippodamia convergens*)
DIPT	Diptera	Flies	Flies not otherwise categorized
DASI	Diptera: Asilidae	Flies	Robber flies
DBBY	Diptera: Bombyliidae	Flies	Bee flies
DSYR	Diptera: Syrphidae	Flies	Hover flies and flower flies
FONA	Formicidae: native	Ants	Native ants—California Harvester Ant (*Pogonomyrmex californicus*), Small Honey Ant/winter ant (*Prenolepsis imparis*), Field Ant (Formica moki), and Odorous House Ant (*Tapinoma sessile*)
FONO	Formicidae: non‐native	Ants	Non‐native ants—Argentine ant (Linepithema humile), Red Fire Ant / RIFA (*Solenopsis invicta*)
HANT	Hymenoptera: Clade Anthophila	Bees	Bees not otherwise categorized: Carpenter, Digger, mining bees (family Andrenidae), leafcutter bees (family Megachilidae), families Colletidae, Halictidae, and Melittidae
HAPI	Hymenoptera: Apis mellifera	Bees	Honey bees
HBOM	Hymenoptera: Bombus spp.	Bees	Bumble bees
HSPH	Hymenoptera: Sphecidae, Crabronidae	Wasps	Thread‐waisted wasps and mud dauber wasps
HVES	Hymenoptera: Vespidae	Wasps	Yellow jackets (*Vespula pensylvanica* and *Vespula vulgaris*), paper wasps (Polistinae spp.), hornets, paper, and mason (all social wasps found in this family)
HEMI	Hemiptera	Bugs	True bugs—aphids, leafhoppers, cicadas, Small Milkweed Bug (*Lygaeus kalmii*), Say's Stink Bug (*Chlorochroa sayi*), and Leafhopper (*Gyponana angulata*)
LEPI	Lepidoptera	Moths	
LERH	Lepidoptera: Rhopalocera	Butterflies	True butterflies—Papilionoidea Skippers—Hesperiidae Butterfly moths—Hedylidae
MANT	Mantodea	Mantids	
NEUR	Neuroptera	Lacewings	
ODON	Odonata	Dragonflies	Dragonflies and damselflies
ORTH	Orthoptera	Grasshoppers	Grasshoppers, katydids, and crickets
PHAS	Phasmatodea	Stick‐insects	Stick‐insects and walking sticks

*Note*: Group codes are assigned based on taxonomic category and examples of specific species included in the grouping code are indicated.

Video data of the plant used in focal monitoring was collected intermittently during the stated focal observation period. For this project, we used a Solar Powered Trail Camera WiFi 4K 30FPS 60MP Dual Lens Game Camera with 1080P@30fps video resolution. This camera is fitted with a Sony IMX458 Starvis image sensor with a 6‐layer glass lens for daytime monitoring. Nocturnal data were collected using the camera's 8‐megapixel F1.6 large aperture night visual lens that features a Novatek 96,670 image denoising element. Prior to this effort, we had used a custom camera trap that required extensive assembly and programmatic operation. The shift to the trail camera was thought to make collection of data easier, and as such was experimental during this effort. Observers stationed the camera approximately 50–75 cm from one of the *B. vanessae* plants in bloom. Videos were set to record for a duration of 3 min in time‐lapse mode. Cameras were serviced every 6–8 h and involved changing batteries and exchanging internal 128 GB SD cards. Data were transferred to a hard drive and a summary log of available data was subsequently generated.

Comparison of focal monitoring data and video recordings involved a summarization of the frequency of flower–insect interactions. Data were reviewed for periods of overlap, and we selected two periods of both low and high insect interactions to use in the comparison. Video footage was reviewed by one experienced observer who is a primary author of this study, and insects were classified according to study groups (Table 1).

### 2.1 Insect groupings

Of the approximately 30 orders of insects, the San Diego Pollinator Monitoring Program (SDPMP) focuses on 18 orders that include flower‐visiting insects and the order Araneae, spiders, with the goal of producing a broad insect assemblage of pollinators of our target plant community, CSS (Table 1). Our insect groupings include major functional insect groups associated with classical pollination syndromes: bees, beetles, butterflies, flies, moths, and wasps (Rosas‐Guerrero et al., 2014; Willmer, 2011). These groupings are not standardized, and while some researchers make further distinctions—for example, discriminating between small and large bees, or flies and long‐tongued flies—we chose to use groupings which would allow for nonlethal insect identification.

**TABLE 2:** Summary of focal observation data, emphasizing the interactions between insect groups and plants and highlights the role of native and non‐native groupings.

Encinitas baccharis (*B. vanessae*)	Male plant	Female plant	Total (male and female combined data)
2023 Bloom period	12 July to 29 Aug (48 days)	31 July to 29 Aug (30 days)	12 July to 29 Aug (48 days)
# of monitoring events/# of days monitored	30/26	16/13	30/26
Total insect pollination interactions observed	1181	194	1375
% of interactions by Honeybees (*A. mellifera*)	60%	54%	59%
Total insect pollination interactions observed (*A. mellifera* excluded)	477	90	567
# of insect groupings observed	11	7	11
Most common insect groups observed	*A. mellifera* 60% Hover flies 14% Native bees 10%	*A. mellifera* 54% Hover flies 26% Beetles 6%	*A. mellifera* 59% Hover flies 16% Native bees 9%

Two large orders were further separated into families: Apidae, Bombidae, Vespidae, and Sphecidae from order Hymenoptera, and Syrphidae, Bombyliidae, and Asilidae from order Diptera. Non‐native European honeybees (*Apis mellifera*) were coded separately from all other bees. *A. mellifera* is native to Europe and is now found on every continent except Antarctica. Brought to the United States for agriculture, it is known to produce negative effects on native bee populations, and on native plants (Iwasaki & Hogendoorn, 2022; Nabors et al., 2018; Travis & Kohn, 2023).

The ant family Formicidae was separated from Hymenoptera and noted as native or non‐native ant species, so that we could discuss the presence and activity of native ants within the context of the California super colony of Argentine ants (*Linepithema humile*). This distinction is important because the vast majority of ant species encountered are invasive *L. humile*, and they are more prevalent in areas with greater human activity (Van Wilgenburg et al., 2010). Finally, we noted spiders (order Araneae) which are not insects and have varied interactions and impacts on plant–pollinator networks, being incidental pollinators, but also predators of pollinating insects, and of plant‐eating insects (Knauer et al., 2018).

Discriminating between species can be challenging during field operations, so we obtained photographs of observed insects whenever possible to confirm identifications through use of our SDPMP photo database and iNaturalist. The three student researchers performing data collection at this site are part of the SDPMP and had approximately 1 year of experience monitoring insect floral visitors at other CSS research sites (San Diego Pollinator Monitoring Program, n.d.). Each observer had 2–3 h of field training with more senior student researchers, and they were given photographic and written references describing the CSS plant community and all insect groupings included in SDPMP monitoring with local species seen in the study area highlighted (Table 1). Given that we did not formally evaluate interobserver error, it is important to note that our study did not include a direct assessment of observer consistency. However, we acknowledge perceived differences in data interpretation based on the comparative results with video recordings. Nonetheless, the uniform training and standardized methods employed among observers aimed to mitigate such discrepancies.

### 2.2 Data summarization and analysis

Data were summarized for male and female *B. vanessae* plants and for each data collection method including total insect visitor activity, number of insect groupings and most common native insect groups observed. Plant and insect diversity was assessed using various metrics. Taxonomic richness, a simple metric, is determined by counting the number of unique groupings present in a sample. We utilized two index metrics for assessing biodiversity. The Shannon–Wiener Diversity Index takes into account both taxonomic richness and the relative abundances of taxonomic groupings within a community. Higher values of H' indicate greater diversity, reflecting both taxonomic richness and evenness in abundance. The Simpson Reciprocal Index (1/D) is used to quantify diversity within a community, with higher values indicating greater diversity. Values close to 0 indicate low diversity, meaning few taxonomic groups dominate the community, and values close to 1 suggest high diversity and indicate that taxonomic groups are more evenly distributed.

Additionally, we incorporated an index value for assessing taxonomic group evenness for male and female plants and for each data collection method. The Shannon Equitability Index, a component of the Shannon–Wiener Diversity Index, focuses specifically on evenness. The Shannon Equitability Index provides insights into how evenly individuals are distributed among taxonomic groups in a community. Values close to 1 suggest a more even distribution of individuals among taxonomic groups, while values closer to 0 indicate a less even distribution. We calculated these three indices for both insect and plant groups. These metrics collectively provide insights into the richness, evenness, and structure of plant and insect communities, aiding ecologists and conservationists in understanding and preserving biodiversity within ecosystems.

## 3 RESULTS

Data collected during this project successfully documented the insect–plant interactions associated with the endangered *B. vanessae* plant at the SDBG. From the start of the plant's 2023 bloom on July 12 until the termination of all six plants' bloom on August 29, we collected focal observation data on 30 monitoring visits over 26 days and acquired collectively over 20 h of video footage (Table 2). The male *B. vanessae* plants were first to bloom, and the female plants began blooming on July 31. Focal monitoring occurred between 9 a.m. and 6 p.m. and camera trap video was collected on each day of monitoring. At the start of the female plant bloom, a second camera was deployed so that video was collected for one male and one female at each visit. Daytime temperatures ranged from about 19° to 30°C with 14 days of clear skies, 11 days with cloud cover, and 2 days were mixed. Data collection methods comparison will focus on five sets of simultaneous focal observation and camera trap video, and two nights of only camera trap video.

### 3.1 Focal observations of insect visitors

Insects from 11 groups were documented interacting with the male flowers of *B. vanessae* and seven groups with the female plants over 10 h total of focal observations. Though the male plants bloomed 18 days longer than female plants and were monitored twice as many days, female plants had far fewer plant–insect interactions, only 17% of the total interactions observed at the male plants. Of the 1375 plant–insect interactions recorded, non‐native honeybees (*Apis mellifera*) comprised approximately 59% of diurnal interactions (Table 2). The most abundant diurnal native insects interacting with both male and female plants were Family Syrphidae (Order Diptera) at 11% of interactions, and Order Diptera (exclusive of Asilidae, Bombyliidae, and Syrphidae), at 8%. Family Asilidae, Vespidae, Sphecidae, and Order Odonata were observed only on male plants. Groups for native bees (Order Hymenoptera: Clade Anthophila) and bee flies (Order Diptera: Bombyliidae) were noted only by focal observation. No unidentified data were noted. Observations included five of the six major functional insect groups categorized in our study associated with pollination syndromes: bees (including *A. mellifera*), flies, beetles, moths, and wasps. Butterflies were not noted.

In assessing diversity based on insect–plant interactions and insect group counts, some notable similarities and differences emerge (Table 3). The male plants of *B. vanessae* consistently exhibit greater taxonomic richness and diversity in both insect assessment approaches compared to female plants. The Shannon Diversity Index and Simpson's Diversity Index are highest for male plants when examining insect group counts (H = 1.86 and 1−D = 0.81, respectively). Female plant–insect interactions inclusive of *A. mellifera* interactions had the lowest diversity value (H = 1.34). However, females display the highest evenness in both insect‐plant interactions (E_H_ = 0.64 and 0.75) and insect group counts (E_H_ = 0.86), indicating a more balanced distribution of taxonomic groups visiting female plants of *B. vanessae*.

**TABLE 3:** Abundance and relative abundance of focal observations of insect–plant interactions for *B. vanessae*.

Insect–plant interactions by grouping	Male plant	Female plant	Total (male and female combined data)
HAPI (honey bees)	704 (60%)	104 (54%)	808 (59%)
DSYR (hover flies, flower flies)	169 (14%)	50 (26%)	219 (16%)
HANT (other bees)	115 (10%)	3 (2%)	118 (9%)
DIPT (flies)	89 (8%)	11 (6%)	100 (7%)
DASI (robber flies)	41 (3%)	0	41 (3%)
DBBY (bee flies)	18 (2%)	11 (6%)	29 (2%)
COLE (beetles)	9 (1%)	11 (6%)	20 (1%)
HVES (yellow jackets)	20 (2%)	0	20 (1%)
LEPI (moths)	12 (1%)	4 (2%)	16 (1%)
HSPH (mud daubers)	3 (0.3%)	0	3 (0.2%)
ODON (dragonflies and damselflies)	1 (0.1%)	0	1 (0.1%)
Total	1181	194	1375

### 3.2 Video observations of insect visitors

Video data were impacted by power consumption requirements of the camera as the full 3‐min files could only be collected when fully charged batteries were replaced in the camera. With low power, the equipment resorted to producing 3–30‐s files, often recorded at different time intervals (e.g., 30 s every minute, or 3 s every 30 s). This ultimately reduced the periods of overtime where there was consistency and overlap across the two observation methods. The comparison between focal observations and video is inherently limited and should be considered as an initial exploration of this topic. We were able to use five 10‐min comparative periods for this review and selected a 10‐min period to summarize activity by nighttime insect visitors. All other data either did not provide comprehensive overlap with the focal observation period or was compromised due to low power.

Daytime camera trap video captured 752 interactions in 11 groups (Table 4). Insects were noted from five of the six major functional insect groups included in our study associated with pollination syndromes: bees (including *A. mellifera*), flies, beetles, moths, and wasps. Butterflies were not noted (Table 5). Four ants (Formicidae: non‐native) and 72 unidentified insects were noted in camera data only (Table 4). The nocturnal insect assemblage could only be captured using the camera trap method. Lepidoptera comprised only 8% of diurnal interactions but 82% of nocturnal data (Table 5). An example of these nighttime insect visitors is available in Video S1. Video S2 provides a diurnal insect sample. While collection protocol in this study did not record the time an insect spent interacting with a flower, it was notable that the moths seen in the nocturnal video appeared to be the same three to four individuals, and they spent much longer periods in contact with flowers than most diurnal insects, with the possible exception of beetles (Coleoptera). Though more specific identification was not possible due to the lack of detail in the video, at least three different types of moth (Lepidoptera) were clearly present using body size and shape to distinguish. Four unidentified flying insects were noted, covering 18% of interactions (Table 5).

**TABLE 4:** Calculation of diversity and evenness values for focal observation of insect visitors measured in two ways, based on the number of insect–plant interactions and subsequently on the number of insect group counts.

	Male plant	Female plant	Total (male and female combined data)
Diversity based on number of insect–plant interactions (including *A. mellifera*)			
Shannon diversity index (H)	1.39	1.34	1.35
Shannon equitability index (E_H_; evenness)	0.58	0.64	0.56
Richness	11	7	11
Simpson's diversity index (1−D)	0.61	0.69	0.62
Diversity based on number of insect–plant interactions (excluding *A. mellifera*)			
Shannon diversity index (H)	1.70	1.35	1.71
Shannon equitability index (E_H_; evenness)	0.74	0.75	0.75
Richness	10	9	10
Simpson's diversity index (1−D)	0.77	0.65	0.77
Diversity based on insect group count			
Shannon diversity index (H)	1.86	1.68	1.83
Shannon equitability index (E_H_; evenness)	0.78	0.86	0.76
Richness	11	7	11
Simpson's diversity index (1−D)	0.81	0.80	0.80

**TABLE 5:** Abundance and relative abundance of insect group detections from five comparisons of focal observation and video recordings.

Insect group	Comparison 1		Comparison 2		Comparison 3		Comparison 4		Comparison 5		Group abundance by method
	07/21/23		07/31/23		08/01/23		08/02/23		08/04/23		
	09:20–09:30		09:05–09:15		12:26–12:36		15:47–15:57		12:02–12:12		
	Focal obs (ML)	Vid	Focal obs (TS)	Vid	Focal obs (TS)	Vid	Focal obs (JK)	Vid	Focal obs (ML)	Vid	
HAPI bees (*A. mellifera*)	5	7	6	–	6	69	116	242	108	188	FO 241 (68%) VID 506 (67%)
DSYR flies (Syrphidae)	11	12	–	22	–	27	–	2	28	16	FO 39 (11%) VID 79 (11%)
DIPT flies	–	–	2	–	2	37	–	–	25	10	FO 29 (8%) VID 47 (6%)
COLE beetles	–	–	–	13	–	–	6	–	–	–	FO 6 (1.7%) VID 13 (1.7%)
HVES wasps (Vespidae)	–	–	–	–	–	17	–	–	2	–	FO 2 (0.6%) VID 19 (1.7%)
LEPI moths	–	–	1	1	2	3	–	–	4	7	FO 7 (2%) VID 11 (1.5%)
DBBY flies (Bombyliidae)	–	–	–	–	–	–	–	–	17	–	FO 17 (5%) VID 0 (0%)
HANT bees (not *A. mellifera*)	–	–	–	–	3	–	9	–	–	–	FO 12 (3%) VID 0 (0%)
FONO ants (non‐native)	–	–	–	4	–	–	–	–	–	–	FO 0 (0%) VID 4 (0.5%)
HSPH wasps (Sphecidae)	–	–	–	–	–	2	–	–	–	–	FO 0 (0%) VID 2 (0.3%)
HBOM bees (Bumblebees)	–	–	–	–	–	1	–	–	–	–	FO 0 (0%) VID 1 (0.1%)
Unidentified	–	1	–	–	–	8	–	12	–	51	FO 0 (0%) VID 72 (10%)
Total daily interactions by method	16	20	9	40	13	164	131	256	184	272	TOTAL FO 353 TOTAL VID 752

*Note*: Initials of each observer are indicated in focal observation (“Focal Obs”) columns. Videos were reviewed by one author, C. Simokat. Start date and time for each comparison period are indicated along with counts and totals for each monitoring method. Counts refer to number of insect interactions with a flower.

### 3.3 Comparative methods

Focal observations yielded a lower number of interactions overall than the camera trap. Comparison of data recorded using both methods simultaneously shows significant variation between individual observers, with one observer capturing 68%–80% of video observations, and another observer recording only 8%–23% (Table 4). Given the observed discrepancies in data interpretation among observers despite standardized training, it is evident that our efforts to mitigate interobserver error may not have been entirely successful. As such, it is important to acknowledge that the degree of error in our study remains unaccounted for. These findings underscore the inherent challenges in achieving complete consistency in observational studies, despite best efforts to standardize methodologies.

While the camera trap documented more than twice the number of interactions, the proportions of the insect groups recorded remained similar for both data collection methods (Tables 5 and 6). Only focal observations noted native bees (Order Hymenoptera: Clade Anthophila) and bee flies (Order Diptera: Bombyliidae). The video observations recorded no instances of these groups. Incorporating nocturnal video collection increased observations of Lepidotpera by 140% overall. Ten percent of camera trap observations included unidentified individuals, and ants (Formicidae) comprised 1%; focal observations recorded neither of these groups. Diversity of insect groups detected with the two different methods were closely similar using all measurements (Table 7).

**TABLE 6:** Relative abundance of functional insect pollinator groups detected by different methods on *B. vanessae* daytime and nighttime.

Insect group	Camera trap (%)	Focal observations (%)
Diurnal		
Bees	67	71
Flies	17	24
Beetles	2	2
Moths	1	2
Wasps	2	1
Ants	1	0
Unidentified	10	0
Nocturnal		
Lepidoptera	82	–
Unidentified	18	–

**TABLE 7:** Comparison of diversity and evenness values for focal observation and camera trap monitoring of insect visitors.

Encinitas baccharis (*B. vanessae*)	Focal observations	Video
Total insect pollination interactions observed	353	752
Shannon diversity index (H)	1.15	1.17
Shannon equitability index (E_H_; evenness)	0.55	0.51
Richness	8	10
Simpson's diversity index (1−D)	0.51	0.52
Most common insect groups observed	*A. mellifera* 68% Hover flies 11% Flies (DIPT) 8%	*A. mellifera* 67% Hover flies 11% Flies (DIPT) 6%

## 4 DISCUSSION

### 4.1 Insect assemblage associated with *Baccharis vanessae*

Our results indicate that *B. vanessae* supports a variety of insect groups and is in turn supported by diverse insect visitors. This insect assemblage is consistent with insect interaction data collected in CSS habitats from all other SDPMP sites (San Diego Pollinator Monitoring Program, n.d.) and with previous studies (Bryant, n.d.; Hung et al., 2015; Moldenke, 1976).

That individuals of many of the same taxonomic groups of insect visitors were noted at both male and female plants is a positive finding as reproductive success for this dioecious population is dependent on the same individuals visiting both males and females to ensure the delivery of pollen. While the male plants had a longer bloom and more plant–insect interactions, this is often found in perennial dioecious species (Barrett & Hough, 2013). Recent studies suggest that the role of pollination by nocturnal moth species is far more important than previously understood, so the finding of significant moth activity in *B. vanessae* is necessary information for conservation of this rare plant (Alison et al., 2022).

The insect functional groups observed at *B. vanessae* align with myophilous, phalaenophilous, and melittophilous pollination syndromes associated with this plant. Excluding *A. mellifera* as it is an invasive generalist, the next most common insect visitors, flies, and small bees, as well as nocturnal moth visitors, are associated with white, cream, yellow flowers with a mild scent and little to no nectar present (Willmer, 2011). Ants were also noted and could be considered part of a “small insect” functional group of generalists, though this is not a regular grouping in our study (Bawa et al., 1985). These syndrome traits are also associated with wind pollination, as mentioned previously, and these results would seem to indicate that *B. vanessae* could be considered ambophilous. Further studies including controlled pollination experiments using insect‐exclusion and wind‐exclusion, and measurement of fruit and/or seed set, would provide more conclusive evidence. Continued investigation would inform conservation strategies for the preservation of this plant.

In our study, we employed camera trap video technology to monitor flower‐visiting insects, aligning with previous research emphasizing the utility of video methods in ecological studies (Azarcoya‐Cabiedes et al., 2014; Bonelli et al., 2020; Curran et al., 2020; Steen & Orvedal Aase, 2011). Our results reveal a diverse assemblage of insect visitors, consistent with the findings of Bonelli et al. (2020), who stressed the importance of combining manual sampling and video observations to comprehensively study flower‐visiting arthropods, echoing our approach. Notably, our dataset encompasses both diurnal and nocturnal insect visitors, with Lepidoptera, primarily moths, accounting for 82% of our nocturnal data. These results indicate the significant role of moths as nighttime insect visitors, aligning with Curran et al.'s (2020) insights into 3D videography's potential for documenting nocturnal insect interactions. While our collection protocol did not record the duration of insect–flower interactions (and sample size was very limited), our initial observations suggest intriguing differences between diurnal and nocturnal insects. The moths observed during the night appeared to engage in longer lasting interactions with flowers, a phenomenon also noted in other studies (Curran et al., 2020). Despite the challenge of precise moth identification due to video limitations, our data clearly indicated the presence of at least three distinct moth types, demonstrating their diversity within the nocturnal pollinator community. Our results also noted the occurrence of unidentified flying insects, accounting for 18% of interactions, suggesting the presence of additional insect visitors that warrant further investigation.

### 4.2 Comparative methods

While limited comparison of the data demonstrated that focal observations underreported insect activity by approximately half, the proportions of common diurnal visitors were similar with both methods, and similar to data collected at other SDPMP sites of CSS habitat (San Diego Pollinator Monitoring Program, n.d.). This indicates that collection is being performed evenly across sites, forming a picture of the significant insect groups involved in pollination of CSS plants. However, it may also mean that monitors are overlooking the same groups with both methods, camera and focal observation, perhaps due to size and speed of some insect visitors. Researchers are given training in insect identification, but many insects associated with pollination services are almost too small for the human eye to see unaided, for example, parasitoid wasp species that range from 10 mm to 0.1 mm (Bryant, n.d.).

There were some important differences in groups observed with each method; specifically, there were no native bees (*Order Hymenoptera: Clade Anthophila*) nor bee flies (*Order Diptera: Bombyliidae*) seen in the camera trap video, both of which are known to be efficient pollinators (Nabors et al., 2018; Wiesenborn, 2003). No unidentified insects were noted in the focal observations. In both cases, these results were likely to be due to methodological limitations. The camera trap allows researchers to scrutinize video, slowing the speed of playback and reversing the video to replay sections. However, with the current camera there is no autofocus feature, and detail can be difficult to discern. Differentiating between flies and bees, and between honeybees (*A. mellifera*) and native bees can be challenging, and this data appears to indicate that focal observations provided better detail than the camera trap to allow differentiation, particularly among visually similar groups (Table 3). As native bees are highly efficient pollinators, data on native bee presence and activity is vital for plant conservation efforts, and therefore improving the ability to perceive detail from camera trap video will be a focus of future study (Nabors et al., 2018).

In performing focal observations, the human observer is limited in their ability to visually track multiple insects, especially those that are fast‐moving or very small. However, an observer is able to move in closer and adjust their angle to view a visiting insect, whereas the camera was restricted to the set field and focus. Observers may be unconsciously biased in favor of tracking more easily recognized targets, resulting in no unidentified insects recorded. The variation in individual observer recording may also be due to bias of the observer toward familiar insects (Table 3).

The camera trap provided evidence of nocturnal insect activity that is difficult to collect through focal observations (Table 2). Recent studies indicate the previously underestimated importance of moth pollination (Alison et al., 2022). Particularly given the significantly longer visits of these nocturnal moths (*Lepidoptera*) observed on video than any of the diurnal insects (Video S1), further investigation and identification of the impact of these species on pollination of *B. vanessae* is warranted.

### 4.3 Limitations and recommendations of the study and potential sources of bias

Camera trap technology is improving quickly and is a promising technology for collecting data on insect pollinators. Several issues need to be addressed to increase camera efficacy and ease of use. While our camera used a solar cell as well as batteries, we encountered issues with the performance of the camera when operating with solar power. This approach is power hungry which could limit the capability of use in remote regions. Evaluation of additional camera equipment is recommended.

The video footage is labor intensive to review and can limit the amount of data that can be incorporated in studies that strictly rely on video review. We recommend exploring expedited methods of detection for video footage. In addition, the perspective and focus of the camera can be a limitation in this type of study, as a wide perspective results in only some blooms being focused in the field of view. We recommend experimenting with both camera orientation and distance from sets of blooms to increase resolution and focus of insects. This is however, challenging, given the spatial orientation of plant blooms.

Finally, observations only occurred at one site which was within a botanic garden which may have influenced the type and abundance of insects present. We recommend conducting this analysis in a natural site of CSS habitat that is both accessible and safe from equipment theft.

## 5 CONCLUSION

Insect pollinators span a wide range of taxonomic groupings, with varying life histories and habitat needs for shelter, nesting, overwintering, and foraging. The results of this study will allow conservation managers of this rare plant to focus their habitat improvement efforts on supporting more targeted groups of insects which have been shown to interact with the plant and may facilitate its pollination. Future investigations could include data collection methods which allow for more specific taxonomic identification of those insect visitors.

**FIGURE 1:**
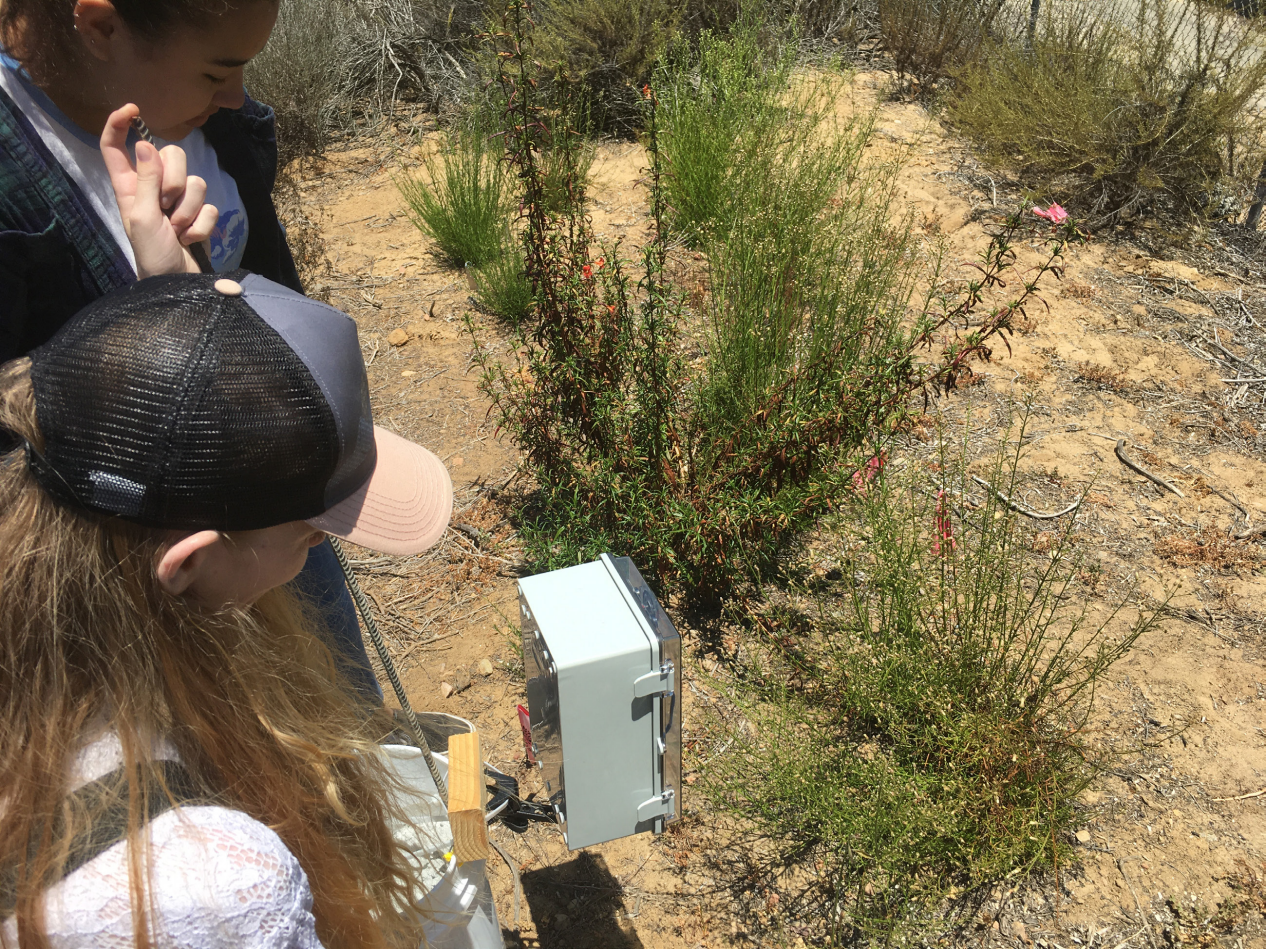
Researchers monitoring *B. vanessae* with original custom camera trap.

This study provides empirical evidence of the value and importance of incorporating multiple monitoring methods in the conservation efforts of endangered plant species. We highlight the limitations of scale associated with focal observations through comparative evaluations with video monitoring. Video technology is progressing rapidly—within 1 year from our preliminary data collection for this study, our researchers were able to replace the cameras we built ourselves with low‐cost complete units with comparable or better features that were far easier to use (Figure 1). While speed of processing remains a short‐term challenge, advancements in object detection and tracking are sure to spur camera monitoring method into the forefront of conservation methodology.

## AUTHOR CONTRIBUTIONS

**Christina Simokat:** Conceptualization (equal); investigation (lead); methodology (equal); project administration (lead); resources (equal); supervision (lead); writing – original draft (equal); writing – review and editing (equal). **Elizabeth L. Ferguson:** Conceptualization (equal); methodology (equal); resources (equal); writing – original draft (equal); writing – review and editing (equal). **Jessica Keatly:** Investigation (equal). **Tyler Smith:** Investigation (equal). **Mia Lorence:** Investigation (equal). **Jasmine O'Hara:** Investigation (equal).

## CONFLICT OF INTEREST STATEMENT

The authors state that there are no conflicts or competing interests related to this research.

### OPEN RESEARCH BADGES

This article has earned Open Data and Open Materials badges. Data and materials are available at https://doi.org/10.1002/ece3.70327.

## Supporting information


Video S1

Video S2

## Data Availability

The data that support the findings of this study are openly available in Dryad at https://doi.org/10.5061/dryad.sbcc2frcz (Simokat et al., 2023). https://datadryad.org/stash/share/GHZ0zpUxwc6LZfksYWElCUTDU01Bi0ppFYBkljCFDHU.
